# Evaluation of long-lasting indoor residual spraying of deltamethrin 62.5 SC-PE against malaria vectors in India

**DOI:** 10.1186/s12936-020-3112-4

**Published:** 2020-01-14

**Authors:** Sudhansu Sekhar Sahu, Sonia Thankachy, Smrutidhara Dash, Krishnamoorthy Nallan, Subramanian Swaminathan, Gunasekaran Kasinathan, Jambulingam Purushothaman

**Affiliations:** Indian Council of Medical Research-Vector Control Research Centre, Puducherry, India

**Keywords:** *Anopheles culicifacies*, *Anopheles fluviatilis*, Deltamethrin 62.5 SC-PE, India, Indoor residual spraying

## Abstract

**Background:**

Deltamethrin 62.5 polymer-enhanced suspension concentrate (SC-PE) is one of the World Health Organization-approved insecticides for indoor residual spraying and was recommended to evaluate its residual activity for determination of appropriate spray cycles in different eco-epidemiologic settings. In the current study, efficacy of deltamethrin 62.5 SC-PE was evaluated against vectors of malaria and its impact on malaria incidence in a *Plasmodium falciparum* hyper-endemic area in Koraput district, Odisha State, India.

**Methods:**

The trial had two comparable arms, arm 1 with residual spraying of deltamethrin 62.5 SC-PE and arm 2 with deltamethrin 2.5% WP (positive control). Comparative assessment of the impact of each intervention arm on entomological (density, parity, infection and human blood index), epidemiological (malaria incidence) parameters, residual efficacy and adverse effects were evaluated.

**Results:**

Both the arms were comparable in terms of entomological and epidemiological parameters. While, deltamethrin 62.5 SC-PE was found to be effective for 150 days in mud and wood surfaces and 157 days in cement surfaces; deltamethrin 2.5% was effective only for 105 days on mud surfaces and 113 days on cement and wood surfaces.

**Conclusions:**

Deltamethrin 62.5 SC-PE had prolonged killing effectiveness up to 5 months. Hence, one round of IRS with deltamethrin 62.5 SC-PE would be sufficient to cover two existing malaria peak transmission seasons (July–August and October–November) in many parts of India.

## Background

Indoor residual spraying (IRS) using insecticide is a key means of malaria vector control worldwide [1]. The World Health Organization (WHO) has recommended 16 insecticides with five varieties of chemical groups (one organochlorine, 8 pyrethroids, 4 organophosphates, 2 carbamates and one neonicotinoid) for IRS based on vector susceptibility, environmental safety, efficacy and cost effectiveness [2]. All the classes of insecticides used for IRS are available in different formulations, such as capsule suspension (CS), wettable powder (WP), wettable granules (WG), and suspension concentrate (SC). Dichloro-diphenyltrichloroethane (DDT) was the first insecticide used in IRS for the Global Malaria Eradication Programme [3] since late 1950s, including India [4]. However, due to numerous concerns, the IRS programme has been failing, leading to re-emergence of malaria as observed elsewhere [1]. Among these, emergence and spread of vector resistance to four classes of insecticides, except neonicotinoids is a serious concern [4, 5].

According to the latest estimates of the WHO, India contributed 67.8% of malaria cases of the Southeast Asia Region (SEAR) during 2017 [6]. A total of 840,838 malaria cases were recorded in the country during 2017, of which 63.4% were *Plasmodium falciparum* cases [7]. Malaria is transmitted by six primary vector species in India: *Anopheles culicifacies*, *Anopheles fluviatilis*, *Anopheles stephensi*, *Anopheles baimaii*, *Anopheles minimus*, and *Anopheles sundaicus* [8]. Among these, the most prominent mosquito species responsible historically for malaria transmission in India is *An. culicifacies,* contributing to 65% of malaria cases in rural areas, followed by *An. fluviatilis* contributing nearly 15% in hilly terrains [9]. To control the vectors of malaria, residual spraying using DDT is a long-standing practice in India [10]. Due to continuous use of DDT, *An. culicifacies* developed resistance to it all over the country [9], resulting in decline of use of DDT in IRS since the last two decades. This prompted National Vector Borne Disease Control Programme (NVBDCP) to switch-over to other classes of insecticides, preferably synthetic pyrethroids, as they are cheap and long-lasting with low mammalian toxicity [10]. Currently, the malaria control programme of India is facing a great challenge, as one of the malaria vectors in the country, *An. culicifacies*, has developed resistance to deltamethrin due to shared mechanisms with organochlorines and widespread use of this class of insecticide in treating the mosquito nets [9, 10]. To tackle the growing threat of pyrethroid resistance in malaria vectors in India and elsewhere [11], industries are manufacturing newer vector control tools, such as insecticide mixtures combining two active ingredients with different modes of action.

Deltamethrin 62.5 polymer-enhanced suspension concentrate (SC-PE) (K-Othrine Polyzone, Bayer Crop Sciences, Germany) is one of the WHO-approved insecticides for IRS [11]. It is a solid active ingredient (AI) formulation dispersed in water, containing 62.5 g of AI deltamethrin per litre, which potentiates the insecticidal efficacy when applied on different surfaces due to the addition of a precise polymer. A recent successful small-scale field study conducted in experimental huts in Tanzania, for evaluating the efficacy of IRS with deltamethrin 62.5 SC-PE against malaria vectors, revealed that the residual efficacy lasted longer with deltamethrin 62.5 SC-PE compared to DDT 75% water-dispersible powder [11].

Further, it was recommended to evaluate the residual activity of deltamethrin 62.5 SC-PE for determination of appropriate spray cycles in different eco-epidemiologic settings [11]. The current study evaluated the efficacy of deltamethrin 62.5 SC-PE at 20 mg/sq m mainly against the susceptible (to synthetic pyrethroids) population of *An. fluviatilis* and its impact on malaria incidence in a *falciparum* hyper-endemic area in India.

## Methods

### Study area

**Fig. 1:**
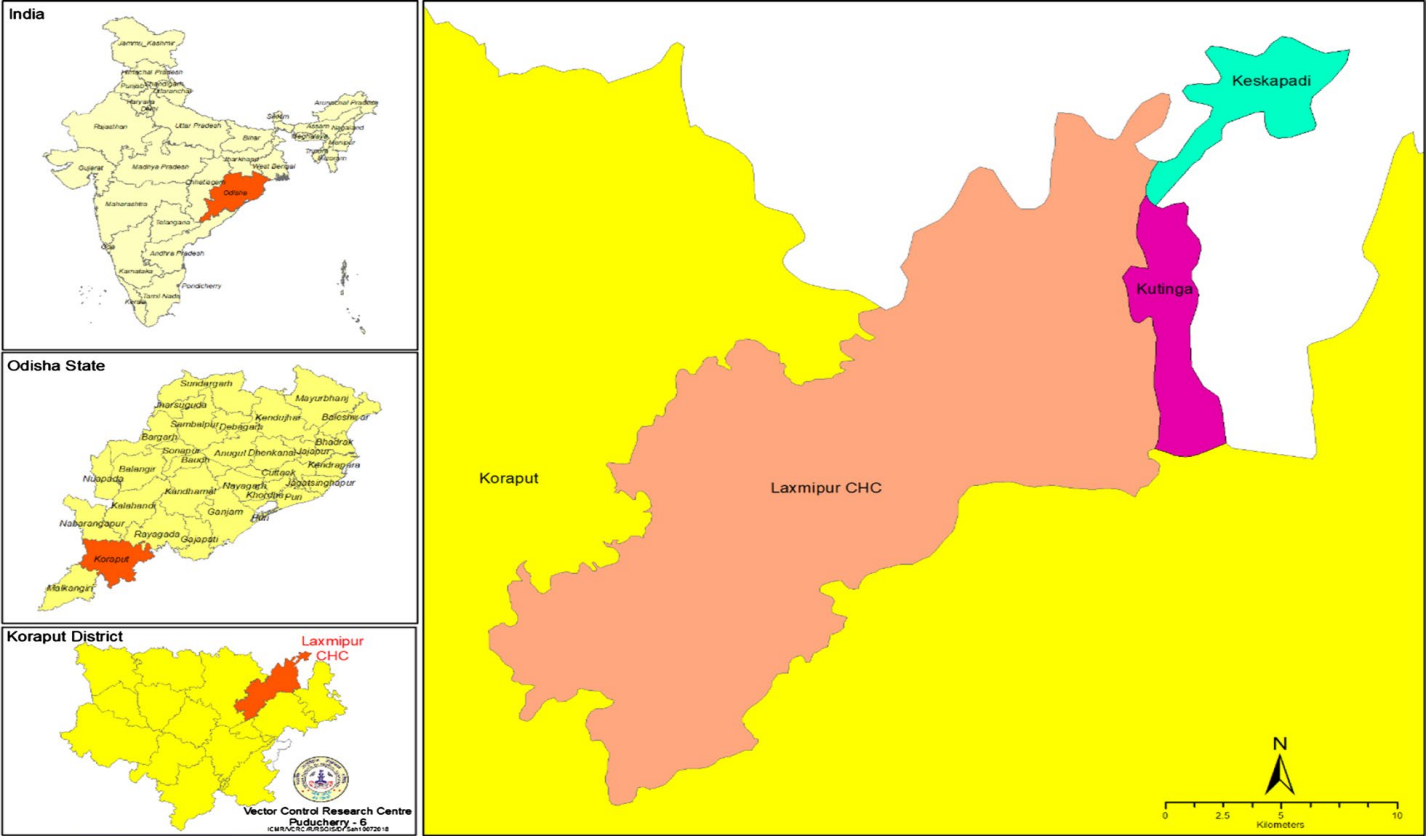
Map showing the study area

The trial was carried out in Laxmipur Community Health Centre (CHC) of Koraput district (18°.8793779′ N latitude and 83°.1683564′ E longitude). Two comparable SCs, i.e., Keskapadi and Kutinga, in terms of eco-epidemiological factors such as population, ecotype, breeding sites, malaria incidence, implementation of personal protection strategies, and abundance of *An. fluviatilis* and *An. culicifacies*, was selected for the trial (Fig. 1). The SC Keskapadi SC had a population of 3921 in 10 villages and Kutinga SC had a population of 5164 in 10 villages. The two SCs were highly endemic for *falciparum* malaria [12]. *Anopheles fluviatilis* and *An. culicifacies* were the major vectors of malaria [12]. While, *An. fluviatilis* is susceptible to deltamethrin, *An. culicifacies* is resistant in the study area [10]. The annual parasite incidence (API) of Keskapadi SC ranged from 83.2 to 132.6 and Kutinga SC from 99.1 to 85.1 from 2012 to 2016 [7]. The distance between the two SCs is about 5 to 10 km. The trial was carried out in 13 villages of which 7 are from Kesakapadi SC (population: 3144, human dwellings: 719, cattle sheds: 395) and 6 from Kutinga SC (population: 3285, human dwellings: 769, cattle sheds: 420). Majority of the houses in the villages have tiled roofing and mud plastered walls. Two rounds of IRS (first round during May–June and second round during October–November) with DDT are being carried out in the district since 1953 [10]. However, in two study SCs, the last IRS with DDT was conducted during May 2016. The second round of IRS with DDT (during October–November 2016) was not conducted in these two SCs, because it was planned to distribute LLINs in the southern districts by the State NVBDCP. From 2009 onwards, long-lasting insecticidal nets (LLINs) have been distributed in the district [13, 14] and during 2017; LLINs were distributed to the entire population of the ten southern districts.

### Study design

The study was a cluster-randomized trial and the unit of intervention was the village. The current evaluation had two arms, arm 1 sprayed with deltamethrin 62.5 SC-PE and arm 2 with deltamethrin 2.5% WP (positive control), which is normally used for IRS in the malaria vector-control programme in the State. The two comparable arms (two SCs) were randomly selected to receive either one of the two formulations of deltamethrin. Accordingly, deltamethrin 62.5 SC-PE was sprayed in Kesakapadi SC and deltamethrin 2.5% WP in Kutinga SC, both having AI 20 g/sq m. The Human Ethical Committee of Indian Council of Medical Research (ICMR)–Vector Control Research Centre (VCRC), Puducherry approved the study protocol. The impact of deltamethrin 62.5 SC-PE on pyrethroid-resistant Indian vector species *An. culicifacies,* was also assessed in this study.

### Spraying of villages

IRS with both the formulations of deltamethrin at the dosage of 20 mg/sq m was carried out in the selected villages during the second fortnight of June 2017 using hand compression sprayers. All the villages in the two arms were given advance information 7 and 3 days ahead. Information Education and Communication (IEC) activities (community meetings) regarding spraying were conducted by the ICMR–VCRC staff with the help of Accredited Social Health Activists (ASHAs) of the respective villages ahead of 3 days of spraying. The spray men were given personal protective equipments like visors, masks, overalls, boots and thick rubber gloves for their general safety. The entire spraying operation was supervised jointly by the research team of the ICMR–VCRC, Field Station, Koraput and staff of the District Malaria Office, Koraput. Villagers were informed to avoid mud-plastering sprayed walls up to 1 year post-spraying. Particulars on spray coverage and reasons for rejection (if any) were recorded.

### Spray coverage and quality

The total number of houses/rooms and cattle sheds covered in both arms during spraying were recorded and calculated concurrently by direct inspection and expressed as the percentage of dwellings/rooms sprayed. Whatman^®^ No. 1 filter papers (15 cm × 15 cm) were fixed on the walls and roofs of the randomly selected holdings (total sampling spots-3; roof-1, and wall-2 in each of the 10 selected houses in each arm) just before spraying and removed after spraying for assessment of spray quality. The insecticide content of filter papers was analysed at Council of Scientific and Industrial Research-Indian Institute of Toxicology Research (CSIR-IITR), Lucknow. The quality of spraying was assessed based on percentage of houses (3 sampling spots in each selected house) with satisfactory insecticide content of active ingredient per sq m.

### Adverse effects

Prior community consent was obtained for conducting interviews. The participants were given clear explanation in local Odia language about the objectives of the interview. Participants were told that they would be at liberty to participate or not in the interview. A questionnaire survey regarding perceived side-effects of insecticide was carried out in two arms among the handlers of insecticides and spray men after the first and last day of spraying, respectively. Heads of the household in two randomly selected sprayed villages in each arm were also interviewed using the questionnaire about side-effects if any and perceived benefits of IRS 1, 15 and 30 days post-spraying following common protocol [15]. Community acceptability was assessed by interviewing the villagers using a structured questionnaire at the end of the study.

### Mud-plastering of houses

The tribal communities have a practice of plastering house walls frequently with cow dung and mud during different local festivals. After IRS, a survey on mud-plastering was carried out at fortnightly intervals in each selected village of the two arms and the percentage of houses/rooms mud-plastered was calculated. Each survey covered 60 holdings in each arm, visiting 6 to 10 houses selected randomly (depending on the total number of villages in a particular arm) in each study village. The size of the sample was decided based on an ordinary mud-plastering of 60% of houses/rooms fortnightly, with 10% error margin and CI with 95%. The mud-plastering survey was stopped when 100% of the houses/rooms were found mud-plastered.

### Entomological evaluation

#### Relative abundance of vector(s) resting indoors and outdoors

In each arm, 6 index villages were selected for collection of day-time indoor and outdoor resting mosquitoes fortnightly from December 2016 to April 2018. Indoor mosquitoes were collected with the help of oral aspirators and flash lights during morning hours (0600 to 0730 h) by hand catch method from 6 human dwellings and 3 cattle sheds in each village, spending 10 min in each station. Outdoor-resting mosquitoes were collected using oral aspirators from 12 artificially made pit shelters in each village, spending 5 min in each pit from 08 to 0900 h. The number of resting mosquitoes collected from the hand catches of indoors and outdoors were expressed as the number per man-hour indoors (PMDI) and outdoors (PMDO).

#### Human landing collections (HLC)

The whole night, mosquito landing collections (HLC) were done from dusk to dawn (1800–0600 h) in two villages, one house in each village in each arm at fortnightly intervals from May 2017 to April 2018. In each village, two adult male healthy human volunteers (one for indoor and other for outdoor HLC) belonging to age group 18–60 years were selected and written informed consent was obtained before conducting HLC. Collections were done both indoors and outdoors to assess landing rate indoors (endophagy) and outdoors (exophagy). The volunteers were told to sleep on a cot with their legs exposed up to knees. The mosquitoes landing on the bait were collected by the insect collectors using oral aspirators and flash lights. Mosquitoes were collected as soon as they settle on the skin and were stored in a paper cup and collected every hour for processing. Collectors worked in 2-h shifts and were rotated indoors and outdoors to prevent bias associated with innate differences in attractiveness of individuals to mosquitoes. Results were expressed as number of landing vector mosquitoes collected per man per night.

#### Processing of mosquitoes

All anophelines collected indoors, outdoors and HLCs were brought to the laboratory, identified to species level using anopheline identification keys [16] and the abdominal condition of the female vector mosquitoes was recorded. Female mosquitoes of each vector species were dissected for their parity status following the dilatation method [17]. The parity was determined from the dilatation of ovarioles. Blood of the freshly fed female mosquitoes collected from indoors and outdoors (resting collections) were processed for identifying the feeding source by agar-gel diffusion method [18] and human blood index (HBI) was calculated.

To determine the vector infection rate, *An. fluviatilis* and *An. culicifacies* obtained from resting (indoors and outdoors) and landing collections were processed using a polymerase chain reaction (PCR) assay. The vector mosquito species collected before and after spraying in both the arms were separated into head-thorax, abdomen parts and dried. These mosquitoes were kept in 1.5-ml microtubes and sent to the laboratory at ICMR–VCRC, Puducherry. They were categorized by species and habitats with respect to study arms (both pre and post) and then pooled to a maximum of 15 mosquito parts/pool. DNA extraction was done from pools of mosquitoes using the QIAamp DNA mini kit (QIAGEN GmbH, Hilden, Germany). Nested PCR assay was done for detecting malaria parasites from the extracted DNA using the primers as described by Snounou et al. [19]. PCR amplification, post-PCR processing and analysis of result are as described by Sahu et al. and Biggerstaff [12, 20]. The parasite infection rate in vector mosquitoes were calculated using the Centers for Disease Control and Prevention (CDC) software PooledInfRate, Version 4.0 for pools of mosquitoes [20].

#### Residual efficacy of insecticide

Since *An. culicifacies* in the study area was resistant to deltamethrin and *An. fluviatilis* was not available in adequate numbers, susceptibility tests and cone-bioassays were carried out using *Anopheles jeyporiensis*, which was susceptible to DDT and deltamethrin. Susceptibility/resistance status of *An. jeyporiensis* to deltamethrin in the study area was confirmed before spraying by conducting WHO adult susceptibility tests [21].

Residual efficacy of the two deltamethrin formulations (deltamethrin 62.5 SC-PE and deltamethrin 2.5% WP) was determined before and after spraying by cone bioassays [22] in selected houses with different surfaces, such as cement walls, mud walls and wood surface. Three houses were selected randomly in one of the selected village of each arm. In a house, two squares marked with pencil, each having area of 0.09 sq m for each surface type. Therefore, four squares from each type of surface in each arm were marked for carrying out the cone bioassay. For selecting the control surface (unsprayed surfaces), 0.09 sq m hard boards were fixed on sprayed surfaces and bioassays were carried out with adequate replicates. *Anopheles jeyporiensis* were collected from 3 to 4 villages (other than index villages) during the morning hours (06.00 and 07.30). 10 blood-fed adult females of *An. jeyporiensis* were exposed for 30 min to each surface. The mosquitoes were removed after the exposure period and kept in paper cups covered with net cloth. Mosquitoes were fed with 10% glucose solution and transported to the ICMR–VCRC Field Station laboratory and kept at 27 °C ± 2 °C and 80 ± 10% RH for 24 h. After 24 h, treated mortality percent was calculated from the number of dead and alive mosquitoes in each replicate for each surface type and recorded in prescribed data sheets. Abbott’s formula was not used, since the control mortality was < 5% in all the cone bioassays. Results were expressed as residual activity of deltamethrin 62.5 SC-PE and deltamethrin 2.5% WP formulations. The inhabitants were requested not to disturb the marked sprayed surfaces by plastering with mud/white washing, etc. The bioassays were done before 1 day of spray, after the first and 7th day of spraying and thereafter every 15 days, using WHO cones, until mosquito mortality dropped below 80%. Whenever mortality in a village or a specific substrate dropped below 80%, bioassays were performed a week later to confirm the low performance. When mortality was found lower than 80% on two consecutive tests, further testing was stopped.

### Parasitological evaluation

#### Malaria prevalence

To determine the malaria prevalence in the study villages, sample blood surveys (SBS) covering 10% of the total population of all age groups, was done in all the selected villages in each arm. The survey was done before intervention and after completion of the study. Surveys were carried out in selected households in each village following systematic sampling method. The residents of the selected houses were screened for malaria infection through collection of blood smears, staining and microscopic examination. Prior consent was taken from the village head and concerned persons from whom the blood samples were collected by finger-prick method. For collecting blood smears from children, consent was obtained from parents. The result of malaria prevalence was expressed as number of positive cases per 100 blood smears collected (slide positivity rate: SPR).

#### Malaria incidence

To record incidence of malaria, active fever surveillance was done at fortnightly intervals from December 2016 to April 2018 in all the selected villages of both the arms by visiting house to house. Villagers with fever/malaria symptoms were screened for malaria infections. Persons found positive for malaria in fever surveillance and sample blood survey were given anti-malaria treatment following national guidelines. The incidence of malaria was expressed as number of positive cases per 1000 population every month (monthly parasite incidence: MPI).

### Data analysis

To compare the vector density between arms 1 and 2 during pre and post-spraying period, Student’s t-test was used. The changes in density of vectors between arms 1 and 2 were compared using two-way analysis of variance (ANOVA). For comparing the relative changes in parity and HBI of both the vectors between the two arms, binomial logistic regression analysis was used. To compare the differences between the pool positive rates during pre and post-spraying, chi-square (χ^2^)/Fisher’s exact test was carried out. chi-square (χ^2^) test was used to compare the corrected mortality of mosquitoes exposed to different sprayed surfaces between two arms. Malaria prevalence between before and after spraying in each arm and between the arms was compared by using χ^2^ test. Logistic regression analysis was carried out to examine the difference in MPI between arms 1 and 2 during the pre and post-intervention periods. Malaria incidence before and after intervention was compared using log-odds ratio interaction test for each arm.

## Results

### Spray coverage

Out of 719 houses and 2231 rooms in the 7 study villages in arm 1, 96.2% (92.0–100.0%) of targeted houses and 93.5% (84.6–100.0%) of targeted dwelling rooms were sprayed with deltamethrin 62.5 SC-PE. Similarly, of the 769 houses and 2404 rooms in the 6 study villages of arm 2, 94.9% (89.3–98.3%) of targeted houses and 90.3% (82.9–95.9%) of the targeted dwelling rooms were sprayed with deltamethrin 2.5% WP. Remaining houses/rooms were not sprayed due to lock and refusals. A total of 395 cattle shed in 7 index villages of arm 1 and 420 in 6 index villages of arm 2 were sprayed with respective insecticide with 100% coverage in both the arms.

### Mud-plastering

In both the arms, no room was mud-plastered up to 1 month post-spraying. After 1 month post-spraying, 6.6% (n = 198) rooms were sprayed in arm 1 and 22.1% (n = 195) in arm 2. After 1.5 months post-spraying, there was a gradual increase in the proportion of mud-plastering of rooms in both arms. 6 months after spraying, the percentage of mud-plastering in arms 1 and 2 was 68.2 and 70.0, respectively. After 8.5 months, 100% of the rooms were plastered in arm 1 and after 9.5 months, 98.7% of the rooms were mud plastered in arm 2.

### Entomological evaluation

#### Species composition

A total of 9882 mosquitoes belonging to two genera were collected in both the arms during the study period. This included 93.6% anophelines and 6.4% culicines. Anophelines comprising 15 species included *An. fluviatilis* and *An. culicifacies,* the principal vectors of malaria and *Anopheles maculatus*, *Anopheles aconitus*, *Anopheles varuna*, and *An. jeyporiensis*, the secondary vectors in India. Among the anophelines, the most abundant was *An. culicifacies* (33.7%) followed by *An. fluviatilis* (15.3%), *An. jeyporiensis* (14.5%), *Anopheles vagus* (13.9%), and *Anopheles subpictus* (13.3%).

In arm 1, a total of 1752 anophelines comprising 14 species were collected pre-spray. The most abundant was *An. culicifacies* (40.9%) followed by *An. fluviatilis* (12.5%). Post-collections yielded a total of 2856 anophelines comprising 13 species with 41.1% *An. culicifacies* and 10.4% *An. fluviatilis*. In the arm 2, prior to spraying a total of 1730 anophelines comprising 14 species were collected. *An. culicifacies* (30.1%) was predominant followed by *An. jeyporiensis* (25.4%), *An. fluviatilis* (19.3%). During post-collections, a total of 2912 anophelines comprising 14 species were collected. *An. culicifacies* (24.2%) being predominant and *An. fluviatilis* was 19.5%.

#### Relative abundance of malaria vectors

Since the density of *An. culicifacies* and *An. fluviatilis* in human dwellings was zero in most of the months during the study period, the indoor-resting density data for human dwellings and cattle sheds was combined.

#### Relative abundance of *An. fluviatilis* resting indoors and outdoors

**Fig. 2:**
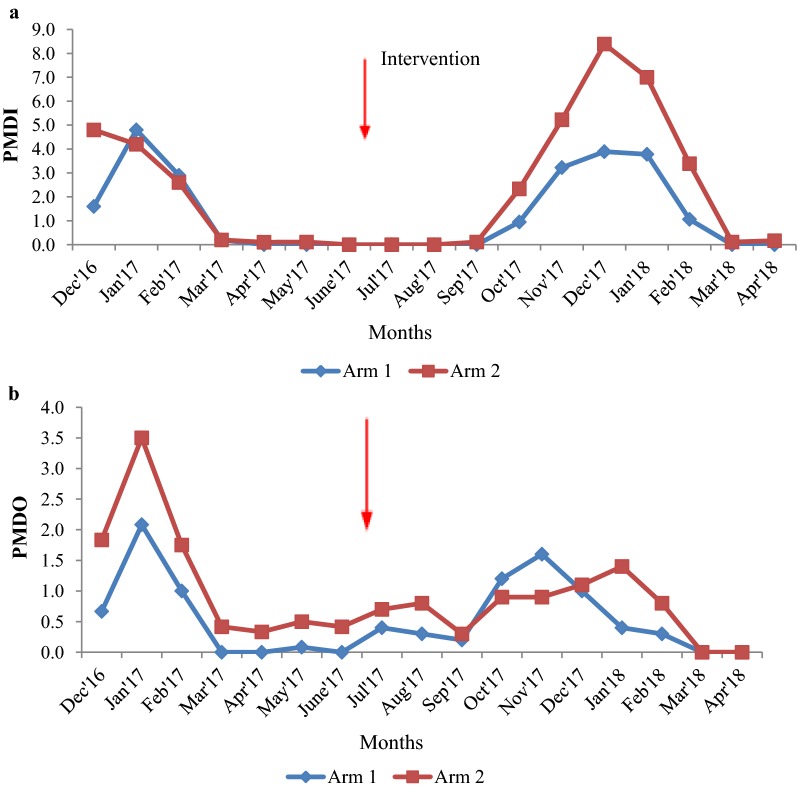
**a**, **b** Relative abundance of *An. fluviatilis* indoors and outdoors prior to and post-spraying

The PMD of *An. fluviatilis* indoors and outdoors prior to and after spraying is given in Fig. 2a, b. There was no significant difference in the average PMDI of *An. fluviatilis* prior to (t = − 0.334, p = 0.744) and after spraying (t = − 1.208, p = 0.243) between the two arms (Table 1). The analysis also showed that the reductions in the PMDI pre to post-spraying did not differ significantly between the two arms (Two-way ANOVA, interaction effect: F = 0.390, p = 0.537). The average PMDO of *An. fluviatilis* did not differ significantly between the arms prior to (t = − 1.266, p = 0.229) and post-spraying (t = − 0.670, p = 0.511). The changes in the PMDO of *An. fluviatilis* pre to post-spraying did not differ significantly between the arms (two-way ANOVA, interaction effect: F = 1.053, p = 0.313).

**Table 1:** Average PMD (indoors and outdoors), HLC and parous rate of *Anopheles fluviatilis* and *Anopheles culicifacies* during pre and post-spraying

Entomological indicators	Species	Arm 1		Arm 2	
		Pre-spraying	Post-spraying	Pre-spraying	Post-spraying
		Average ± SE	Average ± SE	Average ± SE	Average ± SE
PMDI	*An. fluviatilis*	1.36 ± 0.71	1.43 ± 0.57	1.72 ± 0.8	2.97 ± 1.08
	*An. culicifacies*	5.6 ± 1.08	6.41 ± 1.71	4.03 ± 1.15	3.81 ± 1.17
PMDO	*An. fluviatilis*	0.55 ± 0.3	0.54 ± 0.17	1.25 ± 0.45	0.69 ± 0.14
	*An. culicifacies*	0.13 ± 0.08	0.05 ± 0.05	0.09 ± 0.04	0.05 ± 0.05
HLC (indoors + outdoors)	*An. fluviatilis*	0.4 ± 0.1	0.0	0.5 ± 0.1	0.0
	*An. culicifacies*	0.3 ± 0.1	0.0	0.1 ± 0.1	0.0
Parous rate	*An. fluviatilis*	19.5 ± 9.2	15.5 ± 6.2	58.1 ± 9.7	24.4 ± 6.9
	*An. culicifacies*	62.8 ± 7.3	29.3 ± 4.2	62.1 ± 6.7	34.0 ± 4.5

#### Relative abundance of *An. culicifacies* resting indoors and outdoors

**Fig. 3:**
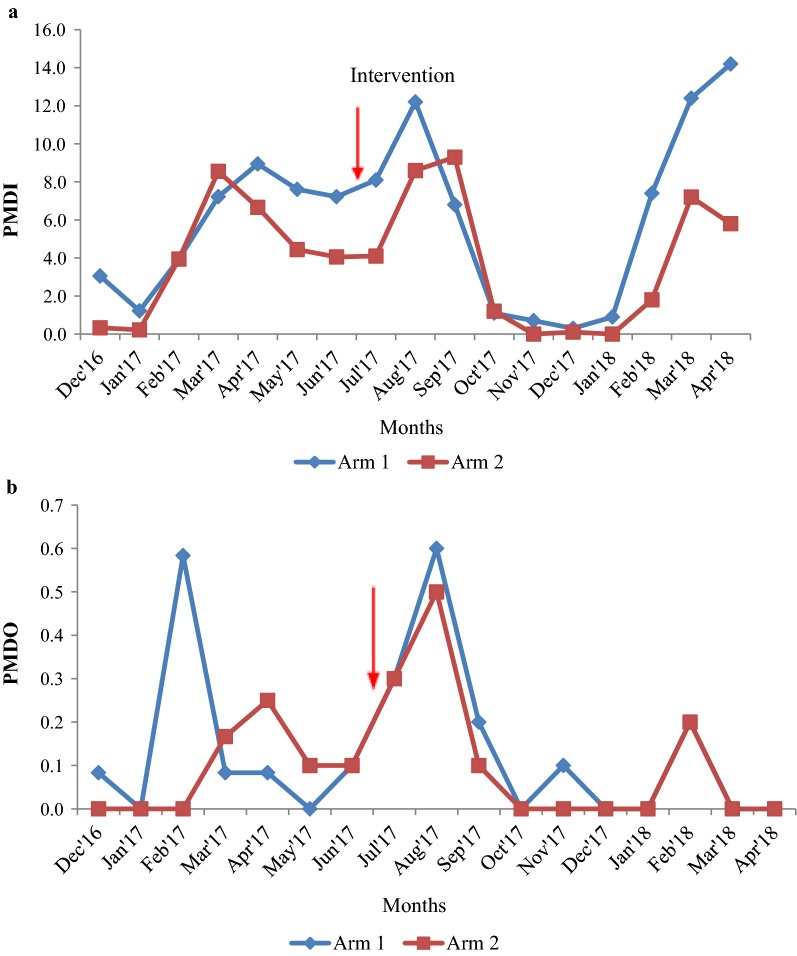
**a**, **b** Relative abundance of *An. culicifacies* indoors and outdoors prior to and post-spraying

The PMD of *An. culicifacies* indoors and outdoors prior to and after spraying is given in Fig. 3a, b. There was no significant difference in the densities (indoors) between the two arms pre (t = 0.984, p = 0.345)/post-spraying (t = 1.257, p = 0.225). The comparative changes in the densities pre to post-spraying did not differ significantly between the two arms (indoors: two-way ANOVA, interaction effect: F = 0.137, p = 0.714; outdoors: F = 0.011, P = 0.918) (Table 1).

#### Relative abundance of An. fluviatilis and *An. culicifacies* in human landing collections (indoors + outdoors)

Before spraying, in arm 1, the density of *An. fluviatilis* and *An. culicifacies* per man per night varied 0.38 to 0.5 (0.4 ± 0.1) and 0.13 to 0.38 (0.3 ± 0.1), respectively. In arm 2, the values varied from 0.38 to 0.63 (0.5 ± 0.1) and 0.0 to 0.13 (0.1 ± 0.1) (Table 1). The density of both the vector species was found to be zero during post spraying collections in both the arms.

### Parous rate

During the pre-spray period, the parous rate of *An. fluviatilis* was 43.4% (n = 212) and significantly reduced to 24.2% (n = 297) during post spraying collection in arm 1 (Wald statistic = 20.338, p = 0.000). In arm 2, the parous rate was 53.4% (n = 326) before spraying and significantly (Wald statistic = 32.515, p = 0.000) reduced to 33.4% (n = 521) after spraying. The reduction of parous rate in *An. fluviatilis* from pre to post-spraying did not show significant difference between arm 1 and arm 2 (Wald statistic = 0.04, p = 0.842; interaction effect: arm × pre-post).

Before spraying, the parous rate of *An. culicifacies* was 61.9% (n = 685) and reduced to 29.9% (n = 968) after spraying in arm1. There was a significant reduction in parous rates between pre and post-spraying period (Wald statistic = 161.29, p = 0.000). In arm 2, the parous rate was reduced to 31.2% (n = 663) from 58.1% (n = 511) during post spraying collection and the reduction was significant between pre and post-spraying period (Wald statistic = 82.9, p = 0.000). The reduction of parous rate in this species from pre to post-spraying did not differ significantly between the two arms (Wald statistic = 1.88, p = 0.17; interaction effect: arm × pre-post) (Table 1).

### Human blood index

The HBI of *An. fluviatilis,* prior to spray was found to be 0 (n = 136) in arm1 and 0.015 (n = 196) in arm 2. After spraying, the HBI of this species was 0.006 in arm 1 (n = 169) and 0.005 in the arm 2 (n = 220) and had not differed significantly from each other (Wald statistic = 0.035, p = 0.852). In the case of *An. culicifacies*, it was low during pre-spraying in both the arms, 0.03 (n = 396) in arm 1 and 0.0 (n = 183) in arm 2. After spraying, the HBI of this species was 0 in both the arms (arm 1: n = 195; arm 2: n = 170).

### Vector infection rate

The maximum likelihood estimate (MLE) of infection rate of *An. fluviatilis* in arm 1 and arm 2 was 0% before spraying and the corresponding values during post-spraying were 0% and 1.01% (CI 0.4–2.2%), respectively. There was no significant difference in the vector infection rate between pre and post-spraying in arm 2 (Fisher’s exact test, p = 0.99). In arm 1, before spraying, the MLE of infection rate of *An. culicifacies* was 0.4% (CI 0.02–1.9%) and after spraying, it was 0.0, showing no significant difference (Fisher’s exact test, p = 0.189). In arm 2, before spraying the MLE of infection rate of *An. culicifacies* was 0.7% (CI 0.04–3.6%) and after spraying, the infection rate was 2.2% (CI 1.2–3.7%); not significantly different (□^□^ = 0.57, p = 0.45) (Table 2).

**Table 2:** The maximum likelihood estimate (MLE) of infection rate of *Anopheles fluviatilis* and *Anopheles culicifacies* during pre and post-spraying

Test particulars	Pre-spraying				Post-spraying			
	*An. fluviatilis*		*An. culicifacies*		*An. fluviatilis*		*An. culicifacies*	
	Arm-1	Arm-2	Arm-1	Arm-2	Arm-1	Arm-2	Arm-1	Arm-2
No. of mosquitoes	4	11	248	132	298	520	962	657
No. of pools	1	1	18	11	25	43	77	52
No. of pool +ve	0	0	1	1	0	5	0	13
Infection rate (95% CI)	0.0	0.00	0.4% (0.02 to 1.9)	0.7% (0.04 to 3.6)	0.00	1.01% (0.4 to 2.2)	0.00	2.2% (1.2 to 3.7)

### Residual efficacy of deltamethrin 62.5 SC-PE and deltamethrin 2.5% WP on the sprayed surfaces

Before spraying, the susceptibility to deltamethrin 0.05% of *An. jeyporiensis* was estimated, using WHO test tubes with four replicates of 100 wild-caught females. After 30-min exposure, 100.0% knock-down and after 24 h holding period, 100.0% mortality were observed, indicating that *An. jeyporiensis* was fully susceptible to deltamethrin.

**Fig. 4:**
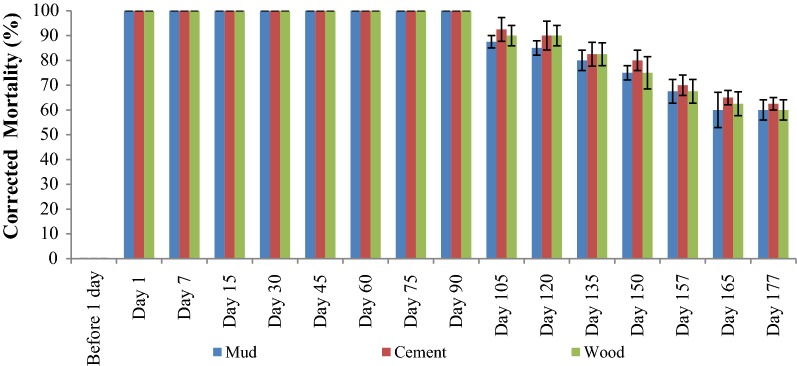
Mosquito mortality in bioassays done on three sprayed surfaces in arm 1

**Fig. 5:**
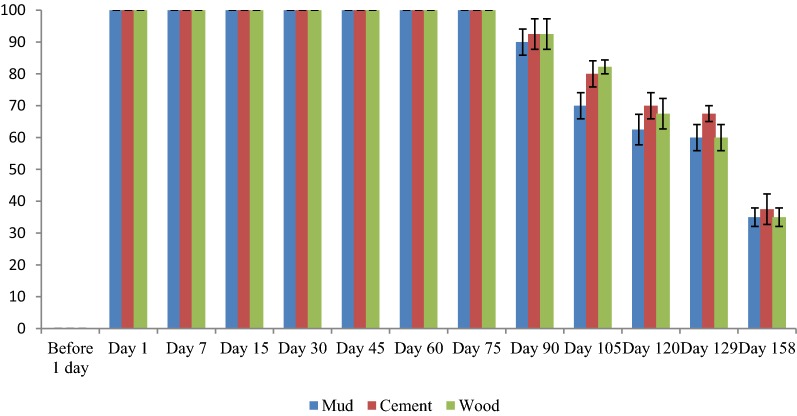
Mosquito mortality in bioassays done on three sprayed surfaces in arm 2

No mortality of *An. jeyporiensis* (n = 120) was observed on wall surfaces in both the arms before spraying, confirming the absence of insecticide deposits on the walls. At 1, 7, 15, 30, 45, 60, and 75 days post-spraying, none of the sprayed surfaces (mud, cement, wood) of both the formulations gave below 100.0% corrected mortality. One-hundred and five days after spraying, while deltamethrin 62.5 SC-PE caused mortality of 87.5, 92.5 and 90.0% in mud, cement and wood surfaces, respectively (Fig. 4), mortality by deltamethrin 2.5% decreased to 70, 80 and 82.5% in the same three surfaces (Fig. 5). At 105 days after spraying, the mortality of *An. jeyporiensis* was significantly (□^□^ = 3.6, p = 0.05) higher in arm1 than that of arm 2 on mud surfaces but not significantly higher in cement (□^□^ = 2.64, p = 0.1) and wood surfaces (□^□^ = 0.95, p = 0.3). At day 113 post-spraying, deltamethrin 2.5% caused mortality of 65.0, 72.5 and 75.5% on mud, cement and wood surfaces, respectively; confirming the residual efficacy of deltamethrin 2.5% lasted up to 105 days in mud surfaces, 113 days in cement and wood surfaces. At 135 days post-spraying, deltamethrin 62.5 SC-PE caused mortality of 80.0, 82.5 and 82.5% in mud, cement and wood surfaces, respectively, and thereafter, mortality decreased to 75, 80 and 75%, respectively, at 150 days post-spraying. In mud and wood surfaces, the insecticidal efficacy dropped below the 80% mortality threshold against deltamethrin 62.5 SC-PE at 150 days post-spraying, which was confirmed 1 week later. It was observed that at 157 days post-spraying, deltamethrin 62.5 SC-PE caused mortality of 67.5, 70.0 and 67.5% in mud, cement and wood surfaces, respectively; confirming the residual efficacy of deltamethrin 62.5 SC-PE on cement surfaces lasted up to 157 days. Bioassays on deltamethrin 62.5 SC-PE mud and wood surfaces were discontinued 157 days post-spraying and cement was discontinued 165 days post-spraying (Fig. 4).

### Residual analysis

The mean ± SE deltamethrin content obtained for deltamethrin 62.5 SC-PE arm was 23.73 ± 3.89 mg/sq m and 33.46 ± 5.12 mg/sq m for deltamethrin 2.5% WP arm. The active ingredient for both the formulation was 20 mg/sq m. The mean deltamethrin content in the filter paper samples was comparable between the two arms (t = 1.52, p > 0.05). Although maximum efforts were made to ensure proper mixing of insecticide and spraying at recommended dosages (20 mg/sq m for both the formulations), there was a variation in initial concentration of insecticides on the sprayed walls between the two formulations, i.e., 23.73 ± 3.89 mg/sq m for deltamethrin 62.5 SC-PE arm and 33.46 ± 5.12 mg/sq m for deltamethrin 2.5% WP arm. While the dose of deltamethrin 62.5 SC-PE was 18.5% higher than the target dose, the deltamethrin 2.5% WP dose was found to be 57.5% higher. There was no relationship observed between the available dosages of two formulations of deltamethrin and the mortality in the bioassays, as with lower baseline content, deltamethrin 62.5 SC-PE performed better in terms of duration of residual efficacy (150 to 157 days) over deltamethrin 2.5% (105 to 113 days).

### Parasitological evaluation

#### Malaria prevalence

Before spraying, 347 (n = 3144) and 339 (n = 3285) blood smears were collected in arm 1 and arm 2, respectively. The average SPR was 21.6% (15.4% to 28.6%) and 28.9% (14.3% to 45.8%), respectively, and there was a significant difference between them (□^□^ = 4.84, p = 0.03). After 10 months of spraying, 325 (arm 1) and 339 (arm 2) blood smears were collected and the average SPR was 0.6% (0.0 to 4.3%) in arm 1 and 0.9% (0.0 to 3.8%) in arm 2 and they were not significantly different (□^□^ = 0.0, p = 0.96). Further, there was a significant reduction observed between SPR of pre-spraying and 10 months after spraying in both the arms (□^□^ = 72.9, < 0.001 in arm 1 and □^□^ = 105.43, p < 0.001 in arm 2).

#### Malaria incidence

**Fig. 6:**
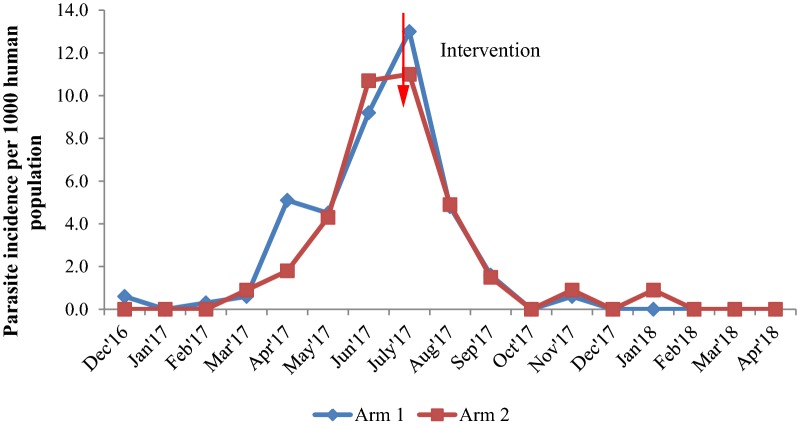
Malaria incidence in arm 1 and arm 2 before and after spraying

Before spraying, the MPI in arm 1 varied from 0.0 to 9.2 with an average (± SD) of 2.9 ± 3.5 and in arm 2, it was varied from 0.0 to 10.7 with an average (± SD) of 2.5 ± 3.9. No significant difference was observed in MPI between the two arms (Wald statistic = 0.618, p = 0.432). After 10 months of spraying, the MPI was reduced in both the arms from 13.0 to 0.0 in arm 1 with an average (± SD) of 2.0 ± 4.1 and 11.0 to 0.0 in arm 2 with an average (± SD) of 1.9 ± 3.5 (Fig. 6). The reduction was maintained at zero level after 4 months of post-spraying up to 10 months with a minor fluctuation (Fig. 6). There was no significant difference observed between MPI over 10 months in two arms (Wald statistic = 0.06, p = 0.8). The reduction in monthly incidence before and after spraying was not significantly different between the two arms (Wald statistic = 0.15, p = 0.69; odds ratio = 1.1; 95% CI 0.67–1.82 interaction effect: arm × pre-post).

### Adverse effects

In arm1, out of 5 handlers of insecticide, 4 (80.0%) felt side-effects such as headache, itching and eye burning which persisted for 24 h, whereas in arm 2, out of 5 handlers of insecticide, 3 (60%) felt headache, itching, eye burning, and head reeling which persisted for 12 h (Table 3). In arm1, out of 16 spray men involved in the spraying, 3(18.8%) felt side-effects such as headache, nausea and itching which persisted for 12 h, whereas in arm 2, out of 16 spray men involved in the spraying, 4 (25.0%) felt headache and itching which persisted for 12 h (Table 3). After 24 h, no adverse effect was reported by the handlers.

**Table 3:** Perceived side-effects of insecticide handlers and spraymen after one day of spraying

Adverse effect	Insecticide handlers				Spraymen			
	Arm 1 (N = 5)		Arm 2 (N = 5)		Arm 1 (N = 16)		Arm 2 (N = 16)	
	Number suffered (%)	Persisted (in h/days)	Number suffered (%)	Persisted (in h/days)	Number suffered (%)	Persisted (in h/days)	Number suffered (%)	Persisted (in h/days)
Bad smell	0	0	0	0	0	0	0	0
Headache	3 (60.0)	24 h	1 (20.0)	12 h	3 (18.8)	12 h	2 (12.5)	12 h
Nausea/vomiting	0	0	0	0	1 (6.3)	12 h	0	0
Itching	4 (80.0)	24 h	3 (60.0)	12 h	3 (18.8)	12 h	4 (25.0)	12 h
Sneezing	0	0	0	0	0	0	0	0
Coughing	0	0	0	0	0	0	0	0
Eye burning	2 (40.0)	24 h	1 (20.0)	12 h	0	0	0	0
Head reeling	0	0	1 (20.0)	12 h	0	0	0	0

After 1 day of spraying, out of 40 heads of the household each from arms 1 and 2 interviewed, all (100.0%) viewed that mosquitoes were killed after spraying and there was no smell of insecticide after spraying the two formulations in their respective villages. Further, the survey conducted at 15 days and 1 month post-spraying reported no adverse side-effects among the households in arms 1 and 2. Hence, after 1 month, the adverse effect survey was discontinued.

At the end of the study, all the 40 household heads interviewed in arms 1 and 2 informed that both the formulations were good insecticide, there was no smell after spraying, no mark on the wall, mosquitoes were killed after spraying and they wanted to use these insecticides in future. This information gives an impression that both the formulations of deltamethrin are acceptable to the community.

### Difference in ease of application

No difference was observed between the two formulations, except preparation of spray solution and quantity of insecticide required for spraying. To cover 3000 population, the required quantity of deltamethrin 62.5 SC-PE for one round of spraying would be 28.8 l and deltamethrin 2.5% WP would be 90 kg. Therefore, for Koraput district with 1.48 million population, the requirement of deltamethrin 62.5 SC-PE would be 14,208 l (142,080 bottles, each bottle contains 100 ml deltamethrin 62.5 SC-PE) and for deltamethrin 2.5% WP, 44.4 metric ton (1480 packets, each packet containing 30 kg of deltamethrin 2.5% WP).

## Discussion

IRS depends mainly on DDT and synthetic pyrethroids for control of malaria vectors in India [9]. After the recommendation of Stockholm Convention on persistent organic pollutants (2004), the use of DDT in IRS programmes in many countries in the world, including India, has been gradually reduced and switched to other insecticides [11, 23]. Considering safety and effectiveness, the use of pyrethroids in IRS in malaria control programmes increased gradually thereafter [24]. In India, among the 8 pyrethroid insecticides recommended by WHO in the framework of WHO Pesticide Evaluation Scheme (WHOPES), deltamethrin formulation was the most accepted and extensively used insecticides and has become very popular [2, 25].

During the last decade, impressive progress has been made in malaria control in India and this has been the result of a large scale-up of vector control measures, mainly the introduction of synthetic pyrethroids in IRS programme sand LLINs [26]. However, reports of insecticide resistance among malaria vectors in many parts of the country threaten these gains and effective malaria prevention as a whole [9, 10, 24]. Failure to alleviate this menace would likely result in an augmented burden of disease, with major cost implications in India. The residual efficacy of IRS insecticides is of vital importance for effective vector control. One of the options is to add a new active ingredient to existing insecticide to form a mixture to delay the spread of resistance. Many industries developed insecticide mixtures with two active ingredients with different modes of action for resistance management. Deltamethrin 62.5 polymer-enhanced suspension concentrate (SC-PE) is one such long-lasting indoor residual spraying insecticide which potentiates insecticidal efficacy due to the addition of a precise polymer [11].

The last IRS with DDT was conducted in the two SCs during May 2016. The second round of IRS with DDT was not conducted in the study villages during October–November 2016, because it was planned to distribute LLINs in the entire ten southern districts of Odisha State. The duration between the last IRS with DDT (May 2016) and the collection of baseline data (December 2016) was 7 months, hence the effect of IRS with DDT might not have affected the study results; as the residual efficacy of IRS with DDT is < 6 months [2]. In the current study, the impact and residual efficacy of deltamethrin 62.5 SC-PE was assessed keeping deltamethrin 2.5% as a positive control against the susceptible population of *An. fluviatilis* and resistant population of *An. culicifacies.* The summarized results indicate that after spraying, no significant difference was observed on entomological parameters, such as indoor and outdoor-resting density, landing density indoors and outdoors, parous rate, vector infection rate, and HBI of both vector species between the two formulations, which suggests both the formulations had similar impact on these parameters. More specifically, the parity rate of both the vector species decreased significantly in both the arms after spraying. The most important finding of the current study are while deltamethrin 62.5 SC-PE was effective for 150 days on mud and wood surfaces and 157 days on cement surfaces, deltamethrin 2.5% was found to be effective for only 105 days on mud surfaces and 113 days on cement and wood surfaces. The rapid mud-plastering observed during 7.5 to 9.5 months in both the arms was due to the *Parabo*, an important festival celebrated by local tribes.

Information on the residual life of the target insecticides is vital to establish the timing and frequency of IRS for protecting populations living in highly malaria-endemic areas during transmission seasons. Inconsistent results were noted in the residual lifespan of two formulations of deltamethrin when natural susceptible anophelines were exposed to different sprayed surfaces, as observed in the current study. Consistent with the results reported in the current study, the residual efficacy of both formulations of deltamethrin sprayed in different surfaces have been found to be the same thresholds (3 to 6 months) suggested in WHO guidelines [27]. The current observation is in line with findings by Maharaj et al. and Dengela et al. [5, 28] that the mortality decreased faster on mud surfaces compared to the other two tested surfaces in both deltamethrin formulations. A study using deltamethrin WG against F1 *An. stephensi* in Iran showed that the residual effectiveness was less than 3 months on cement and mud surfaces [29]. This might be due to the fact that the residual life of insecticides depends upon the type of surfaces [5]. The reason for faster decline of insecticides on mud surfaces as shown in the current study is due to more porous nature compared to smoother surfaces (cement and wood) and dung surfaces as observed in another study [30]. In the study villages, 100% of households were found to have re-plastered their walls by 8.5 months. Despite re-plastering, the significant reduction of incidences of malaria fever up to 10 months post-spraying was observed. It is therefore; clear that the two formulations of deltamethrin spray could be an effective tool to control malaria in these areas if properly applied.

In the present study, when compared to pre-spraying, a significant reduction was observed in malaria prevalence and malaria incidence after spraying in both the arms. Four significant factors that might have influenced the efficiency of house spraying of both formulations of deltamethrin in the study area. First, spray coverage in both study arms was > 90.0%. Secondly, IRS could be effective only if the malaria vectors rest on the sprayed surfaces for an adequate time to pick up a lethal dose [31, 32]. The study areas have been under IRS with DDT since 1953, two target vector species are still endophilic and no changes in their resting behaviour in response to insecticide pressure were observed [12]. Hence, IRS could be an effective tool to interrupt the malaria transmission in the region. Third, insecticide pressure was low due to no use of deltamethrin in routine IRS in the study areas; chances of resistance might be less compared to areas receiving deltamethrin spray regularly. Fourth, deltamethrin might still have an effect of reducing malaria incidence due to its excito-repellent effect on the population of pyrethroid-susceptible *An. fluviatilis* and resistant *An. culicifacies* as observed by Hamusse et al. [32]. In Indian settings, where *An. culicifacies* was resistant to DDT, the same insecticide reduced the malaria incidence considerably [33]. In the current study, the adverse effects reported by the insecticide handlers and the spray men with both the formulations were transient in nature and lasted only up to 12–24 h. The two formulations of deltamethrin are reported to be safe as per their material safety data sheet. The study indicated that both formulations of deltamethrin were well accepted by the community in the sprayed villages.

It is meaningful to judge how the epidemiological trends in both the arms fit with the entomological trends. The declining residual efficacy of IRS in both the arms, as measured using standard wall bioassays, corresponded to reduced entomological effect (parity) but not to reduced malaria incidences. The residual efficacy of both the formulations fell below the 80% cone bioassay mortality threshold after just 105 days post-spray (deltamethrin 2.5%) and 150 days (deltamethrin 62.5 SC-PE) and vector densities increased immediately, but both the interventions appeared to contribute to a reduced malaria incidence for around 5 more months, until the end of the study. The results presented in the current study supports similar results reported from Sudan [34], Tanzania [35], Zimbabwe [36], Zambia [37] and Uganda [38].

Adequate numbers of *An. fluviatilis* mosquitoes could not be collected from the study areas, and so *An. jeyporiensis* mosquitoes that are susceptible to deltamethrin, were used for bioassays in the current study, which was a limitation. Another limitation was that in both formulations the insecticidal dose was found to be higher than the target dose. The difficulty in spraying the target dose in IRS was found to be visible and needs to be addressed when carrying out IRS interventions. There is a need to use the spray equipment with superior technology and quality IRS training for spray operators to overcome these difficulties. Since the trial was carried out in a *falciparum* endemic area where routine vector control measures are being implemented, it was ethically not feasible to include a control arm without any intervention and this was also a limitation. Despite these limitations, the work presented here clearly differentiates the killing effectiveness (150 days for deltamethrin 62.5 SCPE; 105 days for deltamethrin 2.5%) of the two formulations of deltamethrin.

## Conclusions

In India, the wide use of pyrethroids in IRS since 2001 and LLINs since 2009 has probably increased the development and spread of pyrethroid resistance in malaria vectors [9, 10]. Therefore, IRS with deltamethrin should be used with care as part of the management of resistance strategy. Since alternative insecticides for IRS are limited, the findings of this study clarified a place for deltamethrin 62.5 SC-PE as a residual insecticide in malaria control programmes in India. This formulation of deltamethrin sprayed on different surfaces had prolonged killing effectiveness up to 5 months under the prevailing conditions in the study area. One round of IRS with deltamethrin 62.5 SC-PE would be sufficient to cover two existing malaria peak transmission seasons (July–August and October–November) in many parts of India.

## Data Availability

The datasets generated and/or analysed during the current study are included within the article.

## Abbreviations

IRS: indoor residual spraying
SC-PE: polymer-enhanced suspension concentrate
WHO: World Health Organization
WP: wettable powder
CS: capsule suspension
WG: wettable granules
DDT: dichloro-diphenyltrichloroethane
SEAR: Southeast Asia Region
AI: active ingredient
CHC: Community Health Centre
SCs: sub-centres
API: annual parasite incidence
ICMR–VCRC: Indian Council of Medical Research–Vector Control Research Centre
IEC: Information, Education and Communication
ASHA: Accredited Social Health Activist
CSIR-IITR: Council of Scientific & Industrial Research-Indian Institute of Toxicology Research
PMDI: per man-hour density indoors
PMDO: per man-hour density outdoors
HLC: human landing collections
HBI: human blood index
PCR: polymerase chain reaction
CDC: Centers for Disease Control and Prevention
SBS: sample blood surveys
SPR: slide positivity rate
MPI: monthly parasite incidence
ANOVA: analysis of variance
SD: standard deviation
CI: confidence interval
